# Impact of geopolitical risks and innovation on global defense stock return

**DOI:** 10.1371/journal.pone.0312155

**Published:** 2025-02-21

**Authors:** Oana Panazan, Catalin Gheorghe

**Affiliations:** Department of Engineering and Industrial Management, Transilvania University of Brasov, Brasov, Romania; University of Durham: Durham University, UNITED KINGDOM OF GREAT BRITAIN AND NORTHERN IRELAND

## Abstract

This study conducts a comparative analysis of how geopolitical risk (GPR) and innovation impact stock returns in the defense industry based on data from 75 defense companies across 17 countries and 4 continents. With daily datasets spanning from January 1, 2014 to March 29, 2024, wavelet coherence and wavelet phase differences were used to conduct the analysis. The results revealed that innovation had a greater and more pronounced impact during the entire analysis period compared with the influence of GPR events. GPRs exerted an uneven and heterogeneous impact on global defense stocks and had a concentrated impact during events that generated uncertainty. Overall, we found significant time-varying dependence across a large number of companies at different time frequencies. The COVID-19 pandemic did not have a major impact on companies in the defense industry. Further, GPR events led to increased volatility during the Russia–Ukraine war, leading to increased uncertainty. In addition to the dominant role they play in the world defense market, US companies served as a robust hedge, especially from 2021 to 2022. Defense companies in the UK are more sensitive to both GPR events and innovation, followed by companies in Germany and France. Comparative analysis of the scalograms of China reveals a greater influence of innovation compared with GPR events. Thus, diversification opportunities have been extended from the defense industry in China, offering investors a promising way to capitalize on refuge opportunities during periods of disruption. To mitigate the global rearmament trend, we suggest alternative investment opportunities for different time horizons.

## 1 Introduction

In recent years, the defense industry has experienced substantial changes and developments, driven by factors such as geopolitical events and technological progress. The defense industry contributes to economic growth, the consolidation of national security, and innovation [1]. The return and volatility of stock companies in the defense industry are influenced by a combination of governmental, geopolitical, economic, and sector-specific factors [2]. Therefore, investors in this sector need to be attentive to these variables to understand and anticipate market fluctuations.

A primary factor contributing to the development of the defense industry is the increase in global tensions and conflicts. Such aspects are included in the GPR index designed by Caldara and Iacoviello [3], which includes events affecting global peace, such as tensions between states or regions, terrorism, elections, nuclear threats, political unrest, and war. Various authors have presented evidence of the influence of GPR events on stocks. Christofis et al. [4] demonstrated that efficient financial markets quickly absorb the impact of shocks induced by acts of terrorism. Apergis and Apergis [5] examined the effects of terrorism on leading global defense companies and argued that the stock returns of defense industry companies were affected by acts of terrorism. In recent years, studies have advanced the idea that the defense industry was fueled by Russia’s invasion of Ukraine in February 2024 [6,7]. Additionally, the financial effects of the “special operation” began to manifest after the annexation of Crimea in 2014 and escalated with the February 24, 2022 invasion of Ukraine by Russia [8].

Another crucial factor contributing to the expansion of the defense market is rapid progress in defense technologies, especially in the realm of artificial intelligence (AI), unmanned systems, and cybersecurity [9,10]. The positive effect of innovation in the defense sector on economic growth has been established [11,12]. Such innovations enable states to enhance their level of security and operational efficiency. Research has allowed the transition from extensive arsenals to highly innovative and precise weapon systems [1]. Due to the distinct nature of defense organizations, innovation plays a special role as it enables technological spillover from the military domain to civilian applications [13]. The rapid economic development in recent years, driven by AI, contributes to an increase in efficiency and innovation in various industries, including the global defense market. The use of AI in the defense sector includes applications such as data analysis for military intelligence, logistics optimization, development of autonomous systems, and technological innovation [14]. Additionally, opportunities created by AI enable a strategic focus on innovation and technology [15]. Due to the lack of studies on the influence of innovation on the return of defense stocks, our study investigates this issue at the global level.

Previous studies have examined the stocks of aircraft manufacturers before, during, and after the Second World War [16]; the impacts of the terrorist acts in Paris on 13/11 on stocks in the international defense industry [5,17]; the influence of the Arab Spring on the stock performance of global defense companies [18]; the stock performance of US aircraft manufacturers during the Korean Conflict [19]; the effect of Brexit on Defense and Airlines companies from the UK [20]; and the reaction of US industrial stocks to the bombings in Madrid and Bali.

There is ample evidence to indicate that the GPR phenomena affect stocks in various sectors [21–24]. Aslam et al. [25] recommended that the impact of acts of terrorism on stock markets varies depending on the state. Defense stocks are no exception, being affected globally because of GPR [6]. However, the literature on the return of defense stocks is still insufficient. Additionally, the markets have witnessed several geopolitical events in recent years, such as the annexation of the Crimean Peninsula, the Paris Attack, reciprocal sanctions between Russia and NATO countries, the COVID-19 pandemic, the conflict in Ukraine, and the Israel–Hamas dispute. Some studies have examined one of the variables proposed by us in different periods. As an example, the response of the defense market in India to a successful lunar mission was presented by Azmi et al. [26]. Other authors provided evidence of environmental, social, and governance (ESG) effects on defense stocks [27] and the stock market implications of corruption risk in companies in the defense sector [28].

GPR and innovation can influence defense stock returns by increasing government demand for advanced technologies and changing risk perception and market volatility and through competitive advantages provided by innovations and the impact of government regulations and policies [23,24,29]. Geopolitical events directly affect the demand for defense equipment and technologies [11,14]. These events drive technological innovation as governments and companies seek new solutions to respond to emerging threats. Geopolitical tensions and threats can accelerate innovation in the defense sector, as the need to respond to emerging risks creates additional pressure to develop new technologies and strategic solutions. Innovation can mitigate the adverse effects of GPR on defense actions, as more innovative companies are better prepared to face new global challenges [30]. This study aims to address this gap in the literature by analyzing how GPR or innovation could moderate or mediate the effect of the other on defense industry stock returns.

This study contributes to enriching the literature in several aspects. Our study makes a significant contribution by analyzing a previously underexplored interaction: the impact of the dynamic relationship between GPR and innovation on stock returns in the global defense industry. Earlier literature either focused on a specific GPR event [7,31] or considered factors related to innovation [26,30,32]. Second, we study the dynamics of defense stocks at the global level, without limiting ourselves to a small number of companies or the most important ones [6,7,33]. Third, previous studies have revealed that defense stocks were influenced by the Russia–Ukraine war, as demonstrated by Zhang et al. [6] and Bouri et al. [34]. We believe that this war resulted from previous events that began in 2014, with the annexation of the Crimean Peninsulau. Fourth, our study uses advanced techniques to capture short- and long-term changes and highlights how innovation can mitigate the effects of volatility induced by GPR. While other studies often use a single frequency, the wavelet tools allow a simultaneous approach in the time and frequency domains of stock dynamics. As examples, Bossman and Gubareva [35] used quantile-by-quantile regression to process daily stock prices, while Federle et al. [36] used ordinary least square regressions when processing some daily values.

The rest of the work is organized as follows: Part 2 reviews the previous literature. Part 3 presents the data, methodology, framework of the study, and preliminary statistics. Part 4 contains the empirical findings, and Part 5 contains the discussion. Finally, Part 6 presents the conclusions, implications, and potential further developments.

## 2 Literature review

### 2.1 Effect of GPR on defense stocks

The defense industry holds a unique position in any economy as it ensures security and consolidates peace [37,38]. Thus, defense companies, whether public or private, are different from companies in other industries [39], but they share common elements with them [40]. From a structural perspective, defense companies are heterogeneous as they contain a wide diversity of technical competencies and products (http://www.eda.europa.eu). Moreover, international arms trade is widely regarded as one of the most corrupt industries globally [41,42]. Additionally, defense companies belong to the category of policy-sensitive sectors, along with the financial, healthcare, and infrastructure construction sectors [43]. The technologies and materials used, products obtained, and expenditures and budgets allocated are classified or deliberately obfuscated. This highlights why this sector requires further consideration.

GPR events occupy a central place in the existing literature [44–46]. A section of the literature has found various relationships between defense stocks and GPR. Cam [47] found that the defense and telecom industries experienced positive returns following the 9/11 attacks and around the Bali and Madrid bombings. Berrebi and Klor [48] demonstrated that the negative impact of terrorism on Israeli defense companies was 7%, which was two percentage points higher than that of other companies. Although there are numerous studies on the reaction of stocks or other assets to the occurrence of GPR phenomena, surprisingly few refer to defense stocks. For example, Wang and Liu [7] found that geopolitical tensions have affected the returns of defense stocks in China. Similarly, Zhang et al. [6] found significant volatilities of the stocks of most US and European companies around the onset of the conflict in Ukraine. The authors examined daily stock price information for defense and aerospace companies from 10 countries. A more recent study conducted by Bouri et al. [34] tracked returns and volatility for 21 defense companies from six countries. The authors concluded that GPR significantly impacts profitability and volatility, particularly amidst events such as the COVID-19 pandemic and the conflict in Ukraine. Only a few studies have explored the correlation between GPR and the defense sector, indicating that the subject is understudied in the literature.

However, we find evidence of other factors influencing the stock behavior of global defense companies. Koutoupis and Davidopoulos [49] found that the stocks of 17 companies in the defense and aerospace sectors listed on the US stock exchanges were influenced by the dividend policy from 2012 to 2019. Wang et al. [50] stated that China’s military industry has been affected by defense reforms, stock market frictions, and irrational investors. Moreover, other researchers have argued that good news has a greater effect on China’s aerospace and defense industry than bad news and that stock index volatility behaved asymmetrically from 2009 to 2014 [33,51]. Many factors influence the defense sector and its heterogeneous character. The defense industry is characterized by a steady number of firms and minimal turbulence [52]. As a result of the stable and concentrated structure, characterized by a small number of companies entering and exiting the market, an increase in the stock return is possible.

### 2.2 Effects of innovation on the returns of defense stocks

Another stream of the literature has examined the relationship between the defense stock market and technical progress. The determinants influencing the expansion of military companies are different from those of traditional companies [53]. Among these factors, a decisive role is played by technical progress, which allows obtaining cutting-edge technology [54] and technological intensity [55] In general, innovation is a pivotal catalyst for economic growth and the competitiveness of companies [56]. According to Wang and Tang [53], technological innovation is the primary factor that ensures the sustained advancement of defense companies over the long term. The authors concluded that R&D is key to the increase in the stock price of military companies in China. A different perspective is found in the study by Chin [57], which argued that the military role of technology has generated an arms race, which has caused new forms of conflict, although it has led to a reduction in opportunities for war.

From the perspective of innovation, in the defense sector, there is a high percentage of companies with R&D activities and a large number of technological partnerships [58]. Further, there is a high degree of unpredictability in research activities and a long period between the conception and successful technology implementation [30,59]. The particularities of the defense segment are also fueled by the mode of financing, as governments are regulators, financiers, and the main customers. In some states, funding research activity is a necessity due to their geopolitical context [32]. Wang and Tang [53] analyzed Chinese defense companies in terms of profitability, debt management, operating capacity, and R&D. They found that R&D is the determining factor for the expansion of military businesses, and the stock market performance of companies is moderated by the growth rate. We found a different perspective in the study by Hsieh et al. [60]. The authors documented that the aerospace and defense industries have higher standard deviations of innovation capability and high return on earnings. Similarly, external owners or dominant shareholders have the potential to influence management teams toward adopting higher risk–higher return strategies [61]. In the absence of other comparative studies in the literature, we estimate that innovation activity has a considerable influence on the return of defense stocks. In addition, the influence of AI on stocks is vast and diverse, improving analysis, trading, portfolio management, and market security [62,63].

Some studies have revealed the reaction of defense stocks to the emergence of GPR phenomena, especially after the start of the Russia–Ukraine war. In particular, less research is available on innovation regarding the return and volatility of stocks in the global defense industry. Surprisingly, this subject is still insufficiently studied; therefore, there is a need to research the impact of technical progress on defense stocks, following the studies by Béraud-Sudreau & Nouwens [64] and Huo et al. [65]. This study fills a gap in the literature by comparatively investigating the influence of GPR events and innovation on stock returns in global defense companies.

## 3 Data and methodology

### 3.1 Data

This study investigates the relationship between the return of defense stocks and the uncertainty generated by GPR events, as well as the influence of innovation on defense stocks. The study uses relevant data to represent the research phenomena. The data on the ranking of defense companies come from the Stockholm International Peace Research Institute (SIPRI) and have been used in other studies [66–68]. It is one of the most authoritative defense institutes with an open source database of defense spending for 174 countries from 1949 to 2022 [69]. The ranking contains 100 companies, but we found stock market data on 75 of them. The daily stock prices of defense stocks were taken from the investing.com platform [70]. To avoid bias, the sample period spans from January 1, 2014, to March 29, 2024. This period covers several GPR events, such as the annexation of the Crimean Peninsula in 2014; the escalation of the Islamic State; the Paris attack; Britain’s exit from the EU; economic sanctions; mutual relations between Russia and the US, the UK, and the EU; the COVID-19 pandemic; the Russia–Ukraine War in 2022; and the Hamas–Israel conflict in 2023. Similar sampling periods can be identified in the literature [22,24]. For example, Bouri et al. [71] used a sampling period between February 1, 2013, and June 30, 2023, examining the return and volatility spillovers of 21 global aerospace and defence companies. The datasets supporting this study are available in the supplementary data file (see S1 Data File).

We used the GPR uncertainty index proposed by Caldara and Iacoviello [3], which captures geopolitical events in the analyzed states using the matteoiacoviello.com platform [72]. To represent technical progress on defense stocks, we used the MSCI ACWI IMI Innovation Index encompassing large, mid, and small-cap securities across 23 developed economies and 24 emerging economies [73]. It provides a clear picture of innovation, similar to the study by Huo et al. [65]. We calculated the daily return of the stocks of the selected companies using the following equation: Rt=ln(PtPt‐1)∙100, where *P*_*t − 1*_ and *P*_*t*_ are stock prices at day *t − 1* and *t*, respectively. S1 Appendix presents a list of the companies analyzed.

### 3.2 Wavelet coherence

We used the wavelet coherence method to simultaneously analyze the time and frequency of the relationship of the global defense industry with GPR events and innovation. The wavelet coherence framework serves as an alternative to classic time series processing models and has been used in recent years in studying volatility and stock returns [74,75]. Bivariate wavelet coherence presents the relationship between two-time series in both frequency and time domains [76]. This frequency-based approach offers a comprehensive perspective, proving valuable for stock market participants as they make decisions across various time horizons [77,78].

#### 3.2.1 Continuous wavelet transformation (CWT)

Continuous wavelet transformation (CWT) allows us to analyze dynamic relationships between time and frequency variables, which is crucial for capturing the variable and complex nature of the interaction between geopolitical risks and innovation on defense stock returns. A wavelet waves denoted *ψ*(*t*) is a finite differentiable, square time function oscillating in a given time interval that satisfies the following conditions:

$$\int_{‐\infty }^{+\infty }\psi (t)\mathrm{dt},\int_{‐\infty }^{+\infty }{\left|\psi (t)\right|}^{2}\mathrm{dt}=1.$$ (1)

First, the function *ψ*(*t*), called “mother wavelet,” is obtained by translating and scaling on the time axis related functions called “daughter wavelet,” denoted as *ψ*_*τ*,*s*_(*t*), forming the following relationship:

$$\psi_{\tau ,s}(t)=\frac{1}{\sqrt{\left|s\right|}}\psi (\frac{t‐\tau }{s})s,$$ (2)

where *τ∈*ℝ is the location parameter and *s≠0* is the scale parameter such that $\psi (s)=\psi (\frac{t}{s})\mathrm{and}\;\psi (\tau )=\psi (t‐\tau )$. The scaled and translated wavelet has the following form $\psi_{\tau ,s}(t)=\psi (\frac{t‐\tau }{s})$. By shifting and scaling the wavelet function along a time series *x(t)*, the CWT is determined as follows:

$$W_{x}(\tau ,s)=\frac{1}{\sqrt{\left|s\right|}}\int_{‐\infty }^{\infty }x(t)\Psi^{*}(\frac{t‐\tau }{s})\mathrm{dt}.$$ (3)

where Ψ* denotes the conjugated complex form. According to Percival & Walden [79], the reconfiguration of the original time series from the CWT necessitates a condition for the wavelet function *ψ*(*t*) of the time series *x(t)*:

$$\int_{0}^{+\infty }{\frac{\left|\psi (t)\right|}{t}}^{2}\mathrm{dt}<\infty .$$ (4)

A Gaussian modulated wave was used; type Morlet notated *ψ*^*M*^(*t*) is frequently used in financial time series modeling, defined as follows:

$$\psi^{M}(t)=\pi^{‐\frac{1}{4}}{∙e}^{i\omega_{0}t}∙e^{‐\frac{t^{2}}{2}},$$ (5)

where *ω*_*0*_ is the center frequency of the wavelet, and $e^{i\omega_{0}t}$ is the imaginary component of the wavelet function centered at point 0 (*ω*_*0*_*/2π*).

#### 3.2.2 Multivariate wavelet tools

The multivariate wavelet tools used are wavelet coherence, cross-wavelet power, and multiple wavelet coherence. These tools allow us to investigate these relationships at different time scales and identify the driving variables, providing a detailed understanding of how these variables interact over time. The bivariate cross wavelet transformation (YWT) is defined as follows [80]:

$$W^{\mathrm{yx}}=W^{y}{∙W}^{x*},$$ (6)

where *W*^*y*^ and *W*^*x*^ are individual CWT of *x(t)* and *y(t)*, respectively.

$$W^{\mathrm{yx}}(s,\mu )=\frac{1}{\mu }∙W^{y}(s,\mu )∙W^{x*}(s,\mu ),$$ (7)

where *s* and *μ* are frequency and time. Eq (6) can be interpreted as the power of the crossed waves (7):

$$B^{\mathrm{yx}}(s,\mu )=|W^{\mathrm{yx}}(s,\mu )|$$ (8)

According to Torrence and Webster [81], wavelet coherence entails smoothing the cross wave spectrum. The bivariate wavelet coherence of *y* and *x* can be expressed as follows:

$$\delta_{t}^{2}=\frac{{\left|S(W_{t}^{\mathrm{yx}})(s)\right|}^{2}}{S(s^{‐1}{\left|W_{t}^{y}(s)\right|}^{2})∙S(s^{‐1}{\left|W_{t}^{x}(s)\right|}^{2})}$$ (9)

where *S* denotes the smoothing operator. A smoothing procedure is necessary for both cross and individual power spectra as there is a risk of falsely estimating high coherence [82]. A wavelet coherence of 1 indicates a greater similarity between the time series, while a wavelet coherence close to 0 indicates no relationship. The phase of a wavelet indicates any connection between two-time series and can be calculated as follows:

$$\lambda_{\mathrm{yx}}=\tan^{‐1}\frac{I\{W_{t}^{\mathrm{yx}}\}}{R\{W_{t}^{\mathrm{yx}}\}},\;\lambda_{\mathrm{yx}}\epsilon [‐\pi ,+\pi ]$$ (10)

When λ_yx_ is smaller (greater) than $\frac{\pi }{2}$, it indicates that the two series are in phase (respectively antiphase), while the sign of the phase represents the leading variable in the pair of series formed.

**Fig 1:**
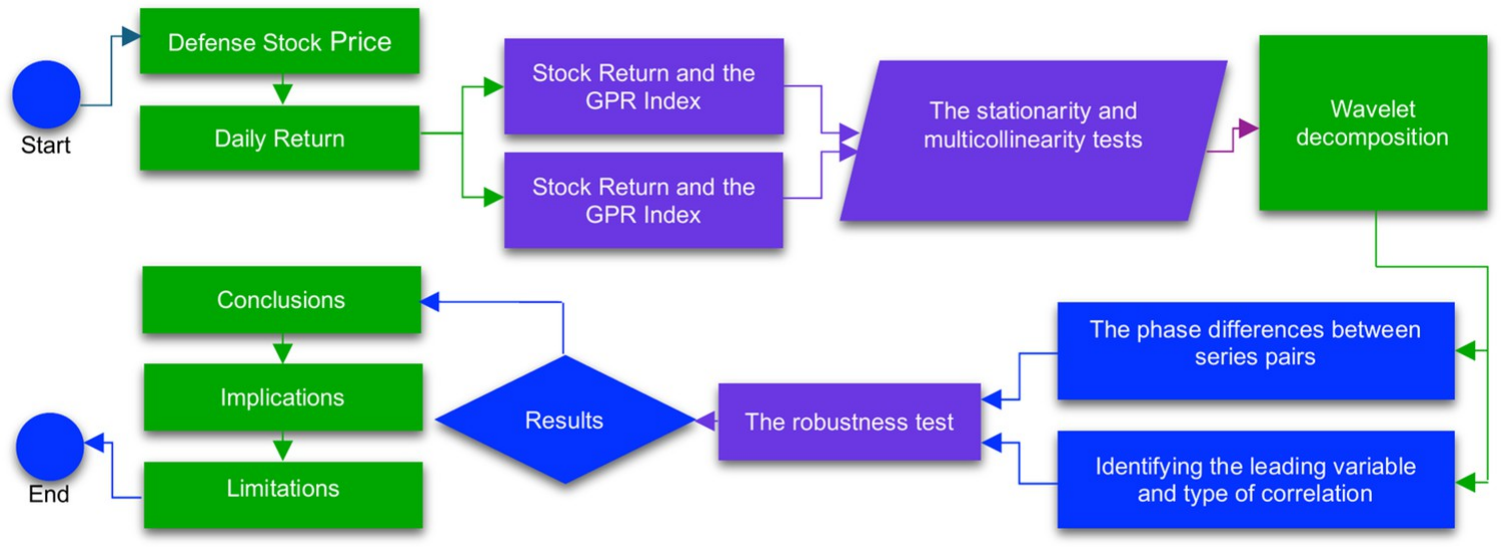
Framework of the study. Source: Own elaboration. Note: This figure describes the conceptual structure of the research.

The study involves going through several stages (Fig 1). In the first step, equal time series are constructed for each company’s stock price return, the GPR index, and the MSCI index. Subsequently, two pairs of series are formed—the first series is between stock return and the GPR index, while the second series is between stock return and the MSCI index. In the second step, stationarity and multicollinearity tests are run. In the third stage, the wavelet transformation is applied between the series pairs. In the fourth stage, the phase differences between the pairs of series are calculated to establish the leading variable and the type of correlation between the variables at different frequencies. Finally, a robustness test is conducted to verify the results.

### 3.3 Preliminary statistics

S2 Appendix presents the statistics for company stocks, the GPR, and the innovation index. Skewness and kurtosis are negative, asymmetric, and leptokurtic. The distribution has heavier tails and a sharper peak, which means that the distributions of series returns are far from normal. The outcomes of the augmented Dickey–Fuller [83] and Kwiatkowski–Phillips–Schmidt–Shin (KPSS) tests reveal that at the 1% significance level, none of the indices have a unit root, indicating that the time series is stationary. Based on the outcomes of these tests reveal that the indices are stationary time series, meaning their statistical properties do not change over time systematically and suggest nonstationarity.

S3 Appendix presents the return and price graphs of the daily series. If most of the series have an increasing rate, then their dynamics oscillated throughout the period (BA, 000065, 000768, 600879, 002268, 600685, GE, ILARSPTA4 = TA, RFL, 7011, FCT, STEG, 2302 and 047810). Moreover, we identify series with a decreasing trend (RR, BAB, CEAD, TKAG, and SRP).

**Fig 2:**
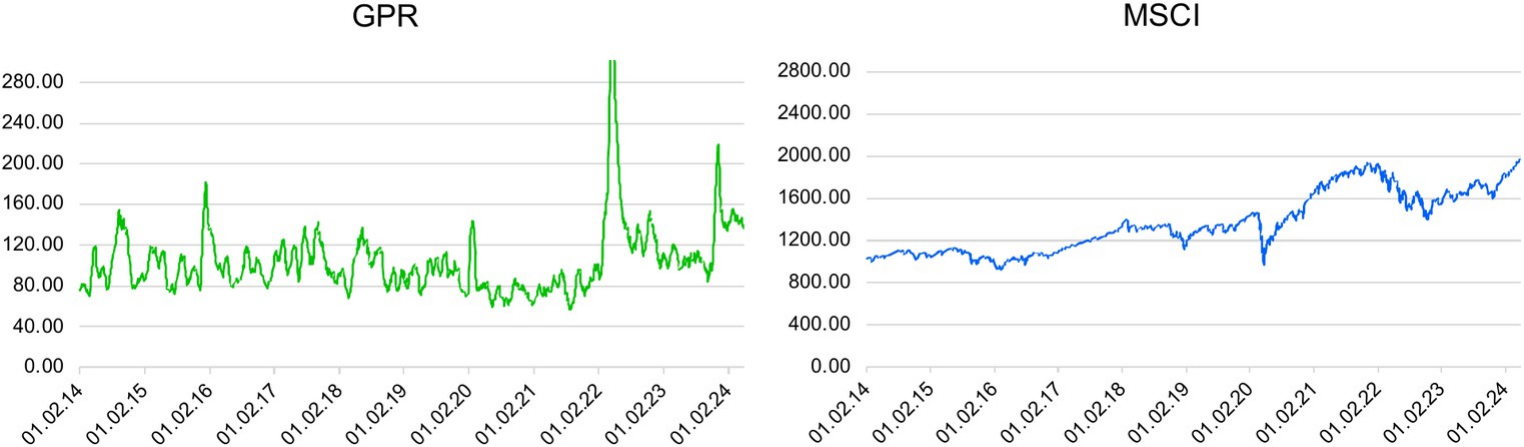
Evolution of GPR and MSCI during the research period. Source: Own elaboration. Note: The X-axis denotes the period from January 1, 2014, to March 29, 2024; the Y-axis denotes index fluctuations.

Fig 2 depicts a higher increase in GRP volatility during Russia’s invasion of Ukraine in February 2022 compared with the annexation of Crimea in 2014. Significant changes are also observed in the case of MSCI in November 2022 and had extreme values in March 2020. Such dynamics are present during extreme events such as the annexation of the Crimean Peninsula, the COVID-19 pandemic, the Russian–Ukrainian conflict, and the Hamas–Israel war. For example, there was high volatility in companies such as LMT, AIR, LHX, BAB, and BA.

The volatility charts of stocks in the defense industry reveal that US companies are adapting to a dynamic and complex environment by focusing on technological innovation, strategic collaboration, and global expansion to maintain and strengthen their market position [64]. The stock prices of European defense companies fluctuated from 2020 until mid2023. For example, CEAD experienced accelerated volatility during the Crimea annexation (2014–2015), the COVID-19 pandemic, and the Russia–Ukraine war (2020–2024). On the contrary, companies in Asia exhibit accelerated and wide-ranging fluctuations, shifting from positive to negative, especially from 2019 to 2023.

## 4 Empirical results

The comparative results are presented in S4 Appendix. The graphs are presented in the form of scalograms, commonly used in the study of stock returns and volatility [84–86]. In each scalogram, the horizontal axis represents the year (2014–2024), while the vertical axis presents the time scale (from 5 to 2,630 days). The colors indicate the intensity of the phase difference, ranging from dark blue (minimum values) to light green (maximum values). The top, central, and bottom of the scalograms correspond to the short, medium, and long term. In the scalograms, blue and green depict regions with weak and strong comovement, respectively.

On each scalogram, there are eight unidirectional arrows. When the arrows point to the right, it signifies that the time series are positively correlated (in phase), whereas arrows pointing to the left indicate that the series are negatively correlated (out of phase). Arrows pointing right and upward (↗) or left and downward (↙) reveal that the initial variable (GPR or MSCI) plays a primary role. Arrows pointing right and downward (↘) or left and upward (↖) suggest a predominant influence from the second variable (stock returns). Arrows pointing upward (↑) or downward (↓) indicate that there is a phase shift of π/2 between the two analyzed series. The area inside the dashed white line, represented by the cone of influence (COI), indicates the region affected by marginal effects, while the area outside the COI indicates insignificant interdependence [80].

**Fig 3:**
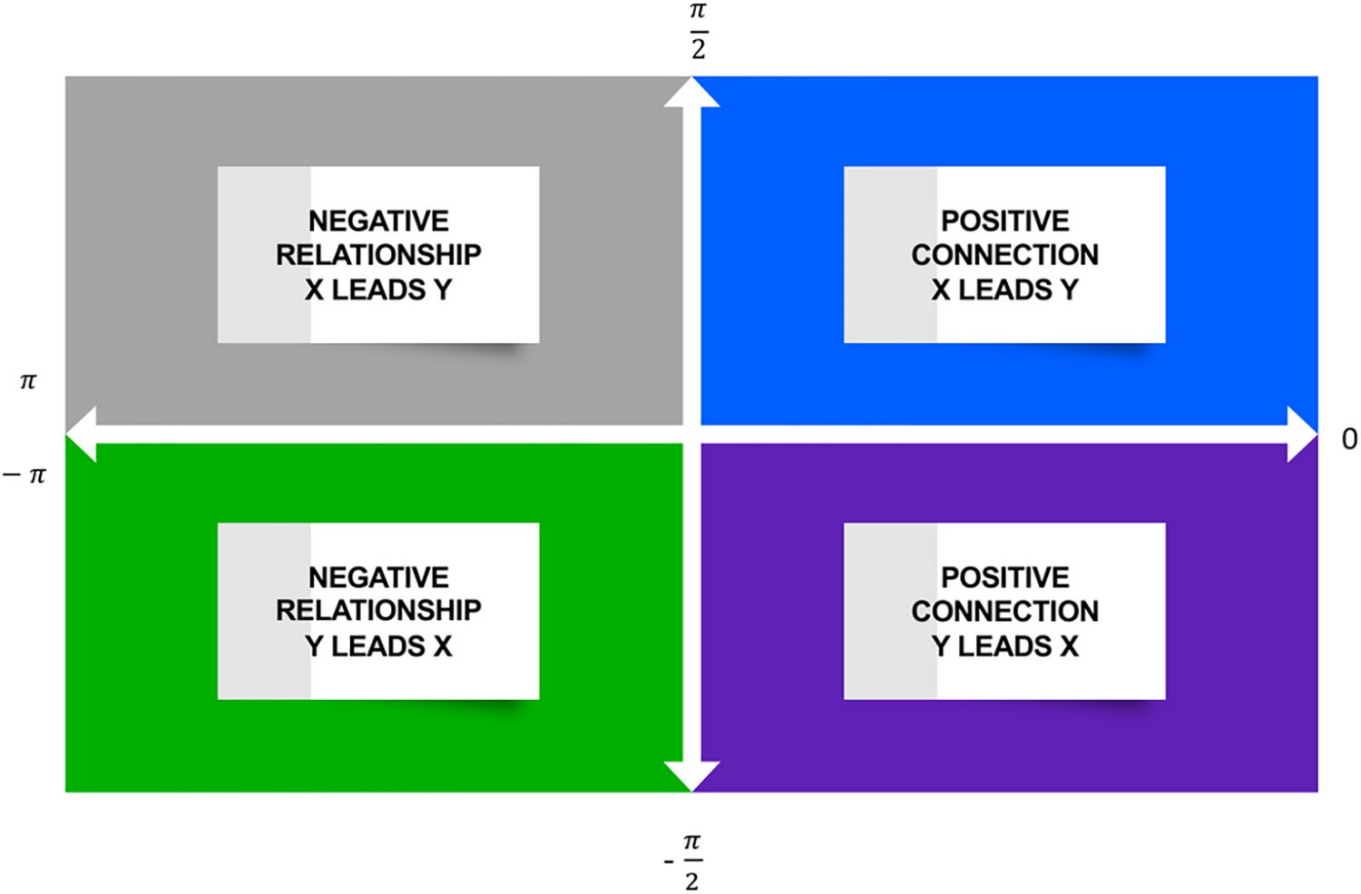
The phase difference matrix. Source: Adaptation after Nasir and He [88].

In Fig 3, *Im* represents the imaginary part of the wavelet transform function. Comovement between *x* and *y* is based on the phase *difference* $\varphi_{xy}(m,n)\in (‐\pi ,+\pi )$. When $\varphi_{xy}\in (0,\frac{\pi }{2})$, it means *x* and *y* have a positive connection, and *x* leads to *y*. If $\varphi_{xy}\in (0,‐\frac{\pi }{2})$, *y* leads to *x*. In contrast, if $\varphi_{xy}\in (\frac{\pi }{2},\pi )$, it denotes a negative relationship between *x* and *y*, and *x* leads to *y*. Finally, if $\varphi_{xy}\in (‐\pi ,‐\frac{\pi }{2})$, it implies *y* leads to *x* [87].

### 4.1 Wavelet coherence for stock return and GPR

For each company, two comparative scalograms related to stock returns and GPR (left) and stock returns and MSCI (right) are presented in S4 Appendix. We find strong correlations between return and GPR in 13 companies (17.3%) from the following countries: the US, the UK, and Germany (3 companies each); France (2 companies); and China and Israel (1 company each). Moderate correlations are found in 41 companies (54.7%), distributed across the following countries: the US (21 companies); the UK and South Korea (3 companies each); China, India, France, and Israel (2 companies each); and Italy, Sweden, Australia, Turkey, Canada, and Norway (1 company each). In 21 companies, the coherence between stock return and GPR is low and insignificant, accounting for 28% of them, originating from the following countries: the US (8 companies), Japan (4 companies), China (3 companies), France, Italy, South Korea, Singapore, India, and Poland (1 company each). Such results provide investors the opportunity to make decisions in proximity to specific GPR events at specific time frequencies.

The Crimean Peninsula annexation in 2014 triggered reactions from 26 companies (34.6%) from the US (NOC, GD, LHX, HII, LDOS, HON, VVX, TDG, PH, TDY, PSN, ETN, and TTMI), China (000065), the UK (BAES and SRP), France (AIR, TCFP, AM, and CEAD), Israel (ILARSP4 = TA), Italy (FCT), South Korea (047810 and 064350), Norway (KOG), and Australia (ASB) from 2014 to 2016. An extremely interesting result is that all these companies reacted in the medium-frequency band. The reaction of companies from the US and Europe stands out. In terms of phase difference, a common reaction of these companies cannot be established, as the direction of the arrows varies from one company to another.

Amidst the COVID-19 pandemic, the reaction of defense companies was not immediate. They reacted with varying delays on the low and medium-frequency bands—38 companies (50.66%) distributed as follows: the US (LMT, NOC, BA, GD, LHX, HON, GE, KBR, TXT, TDG, PH, OSK, J, PSN, ETN, and CW), the UK (BAES, RR, BAB, SRP, and QQ), China (000768, 600879, and 002268), France (AIR, TCFP, AM, SAF, and CEAD), South Korea (047810), Italy (LDOF), Israel (ESLT and RFL), Germany (RHMG, TKAG, and HAGG), Sweden (SAABBs), and Canada (CAE). We find that two aspects were prominent—a delay in the market reaction of defense stocks since the beginning of the COVID-19 pandemic and the low or medium intensity of the fluctuations (they are visible in the graphs in S4 Appendix). This implies that defense stocks were not among the industries deeply affected by the pandemic.

The invasion of Ukraine provoked a stronger reaction compared with all other GPR events during the sampled period. We document the reaction of the following companies from 2022 to 2024 on the medium- or low-frequency band: LMT, NOC, BA, GD, LHX, LDOS, BAH, HON, GE, KBR, TXT, TDG, PH, OSK, TDY, PSN, ETN, MOGa, APH, and MRCY in the US; BAES, RR, SRP, QQ, and MRON in the UK; 000065, 000768, 600879, and 002268 in China; AIR, TCFP, AM, SAF, and CEAD in France; 079550, 047810, and 064350 in South Korea; LDOF in Italy; ILARSP4 = TA, ESLT, and RFL in Israel; RHMG, TKAG, and HAGG in Germany; SAABBs in Sweden; CAE in Canada; HIAE and MAZG in India; KOG in Norway; and ASB in Australia. The proportion of companies in the total sample is 75%, and the intensity of their stock fluctuations is generally moderate.

In terms of frequency (S5 Appendix), companies with strong coherence have high (11 companies) and medium (10 companies) frequencies. Surprisingly, no company exhibits strong fluctuations at high frequency. For companies in which the fluctuation between return and GPR is moderate, the frequency is high (24 companies) or medium (28 companies. Additionally, even in the case of these companies, there is no presence of fluctuation at high-frequency intervals. Thus, these companies serve as potential diversification opportunities, particularly in the short or long run.

### 4.2 Wavelet coherence for stock return and MSCI

Unlike GPR, a more pronounced dynamism is observed between stock return and MSCI (S4 Appendix). We find a strong fluctuation in 34 companies, accounting for 45.3%, distributed as follows: 24 companies in the US; 3 companies in the UK; 2 companies in France; and 1 company each in Canada, Germany, Israel, Japan, and Singapore. We identified a medium level of coherence in 37 defense companies, accounting for 49.3%: 7 companies in the US; 4 companies each in China and South Korea; 3 companies each in France, Japan, and the UK; 2 companies each in Germany, Italy, India, and Israel; and 1 company each in Australia, Norway, Poland, Sweden, and Turkey. Only 4 companies, accounting for 5.4%, recorded low and insignificant coherence: 2 companies in China and 1 company each in the US and India.

Interestingly, fluctuations are much more pronounced compared to GPR events for all analyzed companies, except for BAES (UK), 000065 (China), TCFP, CEAD (France), RFL (Israel), HAGG (Germany), and MAZG (India). The intensity of the fluctuations is higher compared to the values recorded for GPR events, implying that the influence of technological progress in the defense industry is much more pronounced compared with that of GPR events.

Similarly, we observe that around GPR events, the influence of innovation on defense companies increases. After the Crimean Peninsula’s 2014 annexation, significant fluctuations were recorded in the following 51 companies (68%) from 2014 to 2016: LMT, NOC, BA, GD, LHX, LDOS, RYTT, HII, CACI, HON, GE, KBR, SAIC TXT, TDG PH, J, TDY, BWXT PSN, ETN, CW MOGa, APH, BALL, HWM, TTMI, and HEI in the US; RR, BAB, SRP, QQ, and MRON in the UK; 000768, 600879, and 002268 in China; AIR, TCFP, AM, and SAF in France; 047810 and 012450 in South Korea; LDOF in Italy; ESLT in Israel; TKAG in Germany; SAABBs in Sweden; 7011, 7012, and 6755 in Japan; and STEG in Singapore. Interestingly, most of these companies are in the medium-frequency band, but immediate fluctuations also appeared in the high-frequency band. Further, in most of these companies, there is a phase shift of π/2 between the analyzed series, which are positively correlated.

During the COVID-19 pandemic, the innovation activities of the defense companies were pronounced (LMT, RYTT, BA, GD, BAES, LHX, LDOF, AIR, HII, LDOS, AM, ESLT, RR, CACI, HON, RHMG, GE, KBR, SAF, ILARSP4 = TA, SAIC, SAABBs, BAB, 7011, TXT, FCT, VVX, TDG, PH, STEG, OSK, J, TDY, ASELS, TKAG, BAJE, SRP, 7012, 079550, BWXT, QQ, 047810, PSN, ETN, CAE, CW, MOGa, 6755, APH, MRON, ASB, BALL, HWM, TTMI, HEI, 7013). In general, there were fluctuations in the medium-frequency band.

Fully covered scalograms with clouds of arrows, indicating the effect of innovation, which is permanent fluctuations in all frequency bands, were present in the following 21 companies: BA, GD, CACI, HON, GE, KBR, TXT, TDG, PH, OSK, J, TDY, ETN, CW, APH, HWM, and TTMI in the US; AIR and SAF in France; BAB in the UK; and TKAG in Germany. The series are positively correlated in these companies, with stock return playing a major role. Similarly, we observe that technological progress acted as a strong cover against companies from the US and Europe.

## 5. Discussion

This study examines the fluctuation between stock returns and two variables that influence the dynamics of the global defense industry. Here, we examine fresh empirical findings within the framework of ongoing research that national objectives of enhancing national security and maintaining regional stability drive investments in advanced defense technologies. Additionally, the emergence of asymmetric warfare and the need for modernized defense equipment have accelerated the growth of the defense industry. Further, the defense industry benefits from technological progress and generates innovation through its own research structures. As highlighted by Huo et al. [65], the relationship between investment and innovation remains unexplored in the literature.

The 25 years between the end of the Cold War and the Crimean Peninsula annexation in 2014 were characterized by a decrease in European defense budgets, which inevitably led to a reduction in Europe’s defense industrial capabilities [89]. After the Cold War ended, European governments decided to reduce production capacities and preserve them for an extended period. However, during the Crimean Peninsula’s annexation in 2014, reciprocal economic sanctions between Russia and NATO countries led to increased tensions, and, subsequently, the war in Ukraine prompted a rapid reassessment of priorities.

We find a completely different evolution over the entire research period. According to the presented scalograms, the influence of the variables is heterogeneous. Thus, the impact that innovation has on returns is more pronounced compared to GPR events. This conclusion is based on the number of defense companies, the strength of coherence, the position of companies in the SIPRI ranking, the frequency, and the continuity of fluctuations. For both GPR events and innovation, we observe differences from one period to another, among companies in the same country or different countries. Our empirical findings unveil notable diversity between the stock return of the companies and GPR and innovation.

Surprisingly, we observed a different behavior of defense companies toward GPR events and innovation. We confirm the conclusion of Wang and Liu [7] that GPR serves as a predictor of the profitability of the defense sector. While in the case of GPR events, 21 companies (28%) had weak and insignificant fluctuations, only 4 companies (5.4%) experienced it in the case of innovation, which is another finding that supports the difference in the dynamics of the studied phenomena. This means that some defense companies have a greater potential for diversification than others, even when experiencing the same GPR events.

In the presence of GPR events, we detect a stock reaction in the defense sector that is consistent with the finding in the study by Capelle-Blancard and Couderc [40]. Our study affirms the significance of GPR events in the defense industry, in concordance with the results of Zhang et al. [6]. Additionally, our results reveal that the fluctuations had their starting point in the Crimean Peninsula annexation, were modulated by reciprocal economic sanctions between Russia and NATO countries, and were further amplified due to the outbreak of the Russia–Ukraine war. In accordance with the study by Gurdgiev et al. [90], direct involvement in conflicts has a significant positive impact on the performance of defense stocks, but this effect is offset by the negative reaction in subsequent periods. Such results allow for short-term hedging decisions or safe havens for investors. The findings of Apergis et al. [31] indicate that geopolitical events affect the risk profile of defense firms and are less effective in predicting returns. Although the authors considered a large analysis period spanning from 1985 to 2016, there were no comparable global GPR events, such as the COVID-19 pandemic and the invasion of Ukraine. This is why we believe that our results complement both the authors’ analysis period and their findings.

As documented by several researchers such as Gholz and Sapolsky [91] and Mahoney [92], mergers and acquisitions within the sampled companies may explain certain small clouds of arrows found on the scalograms of the involved companies. We studied these events (the results are available upon request). The small clouds of arrows on the high-frequency band offer an in-depth comprehension of the behavior of the time series and can be viewed as normal fluctuations, smoothed out by market self-adjustment [93].

The effect of innovation on the defense industry is more pronounced compared with that of GPR events. The 21 companies that experienced fluctuations across all frequency bands throughout the research period offer a permanent hedging potential. According to Chen et al. [27], ESG objectives in US airlines are an innovation strategy applied as a countermeasure throughout the COVID-19 pandemic. We extend the findings of Chen et al. [27] to defense companies in Europe and China. We also believe that the scenario advanced by Hsieh et al. [60] that there is a relationship between innovation and the performance of defense companies is feasible. A possible explanation is that the dynamics of technical progress in the last decades, accelerated by AI, gives legitimacy to innovation companies, making it an attractive investment option for both individuals and institutional investors.

The COVID-19 pandemic influenced the returns of a total of 23 companies (30.6%) in the following countries: 8 companies in the US; 3 companies each in China and France; 2 companies each in the UK and Israel; and 1 company each in Germany, Norway, Sweden, India, and Italy. The stock reaction of these companies was differentiated across the medium-frequency band, with a few exceptions. Thus, we extend the diversification opportunities in the defense industry in China, as proven by Bouri et al. [34], to other countries, including companies in the US.

US defense companies are central players in the global defense market. Through comparative analysis of the influence of the variables on US companies, we observe that innovation has a stronger impact compared with GPR events. For instance, the Russia–Ukraine war, the most significant event since World War II, did not trigger reactions from all US companies. Given the strong relationship between returns and innovation in US companies, a certain dependency is created for other companies not only in the supply area but also in R&D. We believe that the phenomenon observed by Kleczka et al. [94] at the European level can be extended to other regions globally. We observe that US companies served as a robust hedge, particularly from 2021 to 2222, which is consistent with the findings of Tzeremes [95]. Moreover, we notice a shift in direction compared with the findings of Meijer [96]. The author suggested that in the postCold War period, there was a shift in power toward Europe, driven by transformations in the EU defense industry, which does not align with our results.

The reforms in the Chinese defense industry in recent years aim to bring Chinese defense companies to the forefront of global technological innovation standards [64]. The results of the study by Béraud-Sudreau and Nouwens [64] are confirmed in our scalograms as innovation significantly influences companies in China. A comparative analysis of the scalograms of China reveals a greater influence of innovation compared to GPR events. Therefore, defense companies in China provide opportunities for diversification against GPR events, as noted by Bouri et al. [34]. Our findings indicate that the defense sector in China offers investors a promising way to navigate these challenges and capitalize on refuge opportunities during periods of disruption.

The reaction of defense companies in Europe to the Crimean Peninsula annexation is not as pronounced as that of defense companies in the US, China, or Israel. Moreover, we observe significant differences within Europe. Companies in the UK are more sensitive to both GPR events and innovation. This is followed by companies in Germany and France. In the UK, the influence of Brexit as a result of the June 23, 2016 referendum on defense stock returns is notable. Moreover, in Europe, the time-varying correlation between GPR and the sampled companies implies that the hedging and refuge attributes vary across different periods. The results seem logical because although efforts to build a European defense system intensified after 2013, industrial structures and defense industry funding remained predominantly national [97].

Several previous studies on stock volatility or returns have used coherence levels and wavelet phase differences to define the relationship between two time series [56,98–100]. In the case of comovements between return and GPR, a similar behavior cannot be established; the situation changes when we examine the relationship between return and innovation. At the level of strong coherences, a phase shift of +π/2 is observed, offering limited hedging opportunities for portfolio investors [88].

International financial and economic penalties have emerged as a solution to the escalation of GPR events. We confirm two pieces of evidence presented by Conlon et al. [8] regarding the effect of sanctions on stocks. First, we find that the annexation of Crimea in 2014 affected the global defense industry, but in a differentiated manner. Second, reciprocal sanctions imposed by Russia on one side and the UK, the US, and the EU on the other side can explain the small clusters of arrows on the scalograms of some companies in low-frequency bands. The last major GPR event in the research period is the ongoing Israel–Hamas conflict that began in October 2023. The results reveal a local effect on defense stocks. We did not observe a significant influence on defense companies, except for those in Israel. Additionally, our results contribute to avoiding herd behavior caused by the occurrence of GPR events [101].

### 5.1 Robustness tests

The large number of companies, the connections between them, and the presence of multiple companies in a country made us conduct robustness tests. Additionally, the spillover effects that transfer the advantages obtained through research activities to another company in the defense sector might have influenced the results [102]. We used the nonlinear Granger causality robustness test to capture nonlinear causal relationships between variables, confirming the robustness of identified relationships. This test allows us to distinguish true causal relationships from mere correlations, further validating our results [103–106]. To capture the causality of the studied variables, we utilize the integrated nonlinear Granger causality test introduced by Diks & Panchenko [107].

If *X*_*t*_ and *Y*_*t*_ are two stationary time series, *Y* Granger causes *X* if the past and present values of *Y* hold supplementary information regarding future values of *X*. If F_y,t_ and F_x,t_ are the information samples including the past observations of *Y*_*t*_ and *X*_*t*_ before time t + 1, then „~” denotes equivalence in distribution. Thus, the series *Y*_*t*_ Granger causes *X*_*t*_, when:

$$(X_{t+1},\ldots ,X_{t+k})|\left(F_{\mathrm{Y,t}},F_{\mathrm{X,t}}\right)∼(X_{t+1},\ldots ,X_{t+k})|F_{\mathrm{X,t}}$$ (11)

where k ≥ 1 is the predicted limit, and k = 1 compares the one-step forward conditional distribution of *X*_*t*_ with the past and current values of *Y*_*t*_. If the delay vectors are considered:

$$Y_{t}^{\mathrm{Ly}}=\{Y_{t‐L_{y}+1},\ldots ,Y_{t}\}$$

$$X_{t}^{\mathrm{Ly}}=\{X_{\mathrm{t‐}L_{y}+1},\ldots ,X_{t}\}$$

$$\left(L_{y},L_{x}\geq 1\right)$$ (12)

The null hypothesis posits that prior observations of $Y_{t}^{\mathrm{Ly}}$ contain extra information regarding *X*_*t*+*1*_ compared with $X_{t}^{\mathrm{Lx}}$:

$$H_{0}:X(t+1)|(Y_{t}^{\mathrm{Ly}};X_{t}^{\mathrm{Lx}})∼X(t+1)|X_{t}^{\mathrm{Lx}}$$ (13)

Next, we add the time index and consider that *Lx = Ly = 1*. If the null hypothesis is true, then *Z*’s conditional distribution given by (*Y*, *X*) = (*y*, *x*) is assumed to be the same with *Z*, *Y = y*. Regarding the reports of the common distributions, Eq (13) can be altered such that the common probability density function f_Y,X,Z_(y,x,z) must satisfy the following relationship:

$$\frac{f_{\mathrm{Y,X,Z}}(\mathrm{y,x,z})}{f_{X}(x)}=\frac{f_{\mathrm{Y,X}}(\mathrm{y,x})}{f_{X}(x)}∙\frac{f_{\mathrm{X,Z}}(\mathrm{x,z})}{f_{X}(x)}$$ (14)

According to Eq (14), (*Y*, *Z*) is independent of conditions given *X = x* for each fixed value of *x*. The null hypothesis becomes:

$$P\equiv E[f_{\mathrm{Y,X,Z}}(\mathrm{y,x,z})f_{X}(x)‐f_{\mathrm{Y,X}}(\mathrm{Y,X})f_{\mathrm{X,Z}}(\mathrm{X,Z})]=0$$ (15)

The local density estimator is denoted as *d*_*γ*_, i.e., the random vector with variation *γ* to *γ*_*i*_, by

$${\hat{f}}_{\gamma }(\gamma_{i})=\frac{{\left(2\delta_{n}\right)}^{‐d_{\gamma }}}{\left(n‐1\right)}\sum_{\mathrm{j,j}\neq i}I_{\mathrm{ij}}^{\gamma }$$ (16)

where $I_{\mathrm{ij}}^{\gamma }=I(\|\gamma_{i}—\gamma_{j}\|<\delta_{n})$ with indicator function I(⋅) and bandwidth *δ*_*n*_ as a function of sample size *n*. The test statistic may be expressed as follows:

$$T_{n}(\delta_{n})=\frac{n‐1}{n(n‐2)}∙\sum_{i}\left({\hat{f}}_{\mathrm{Y,X,Z}}(Y_{i},X_{i},Z_{i}){\hat{f}}_{X}(X_{i})‐{\hat{f}}_{\mathrm{Y,X}}(Y_{i},X_{i}){\hat{f}}_{\mathrm{X,Z}}(X_{i},Z_{i})\right)$$ (17)

For *Ly = Lx = 1*, when ${\delta_{n}=\mathrm{Cn}}^{‐\beta }(C>0,\frac{1}{4}<\beta <\frac{1}{3}),$ statistical test T_n_(δ_n_) satisfy:

$$\sqrt{n}\frac{\left(T_{n}(\delta_{n})‐q\right)}{S_{n}}\overset{D}{\to }N(0,1)$$ (18)

where $\overset{D}{\to }$ represents the convergence of the distribution, and S_n_ is a variance asymptotic estimator of T_n_(∙). The one-tailed test is performed using Eq (18) [108].

The nonlinear Granger test examined the relationship between GPR, MSCI, and stock prices in the defense industry within a temporal frequency framework (S6 Appendix). There is significant evidence suggesting that the lagged values of the series Granger cause GPR by 10.7%, while the lagged values of the series Granger cause MSCI by 32%. Due to space, the extended results are not reported but are available upon request.

## 6. Conclusion and implications

This study investigates the impact of GPR events and innovation on stock returns in the global defense industry, considering various investment horizons. We present a comparative analysis over a considerable research period (2014–2024) using daily data. When events that cause uncertainty occur, defense stocks attract the attention of investors. To verify this proposition, the relationship between GPR events and return was considered. Additionally, the relationship between returns and innovation, another determinant variable for the defense industry, was also considered. In particular, the progressive impact of technological progress on the defense industry is a less studied segment of the literature, requiring further investigation. Our findings suggest that the advantages of diversification and risk hedging across global defense companies are pronounced, mainly over the medium to long term, and vary among companies. The results reveal that the defense industry served as a strong hedge against GPR from 2018 to 2022 for companies in Europe, while technological progress was a hedge alternative for companies in the US and China. Moreover, companies in China offer strong diversification advantages against GPR. Conversely, on a global level, companies in the US and Europe act as significant hedges during GPR events. In addition, using the nonlinear Granger test, we demonstrate that there is no significant causal relationship between the GPR–stock return and innovation–stock return pairs. The inclusion of innovation in the study of stock returns offers decision-making alternatives to market participants. They should closely examine the dynamics of innovation and how the results are assimilated by the defense industry to inform decisions based on market conditions.

### 6.1 Implications and limitations

Our empirical results reveal different intensities of interaction between the analyzed variables under different defense companies and time intervals. Investors can use this information to make informed decisions regarding hedging or diversification transactions when GPR events occur. Furthermore, innovation in the defense sector offers greater investment opportunities, and investors should monitor companies that invest in R&D as they could generate superior long-term returns. Thus, to mitigate the resurgence of global rearmament, defense companies should focus on innovation and draw investors’ attention by offering financial rewards. Innovation in the defense sector also provides policymakers with decision-making options. When formulating security policies and setting defense budgets and oversight mechanisms, policymakers should include assessing the potential impact of geopolitical uncertainties on the market. Moreover, governments can implement policies to support defense companies through fiscal and financial incentives to stimulate innovation and attract investment. Researchers can further explore other macroeconomic variables or industry-specific factors that influence the performance of defense companies. Furthermore, this study provides a basis for developing empirical models to integrate these variables into a robust risk management strategy.

This research has several limitations that future studies may address. First, our analysis focused on only two major variables—GPR and innovation—ignoring other macroeconomic variables that could significantly impact stock returns. Second, we used representative indices to measure GPR and innovation; future research could use specific thematic indices or more detailed data sources to explore the relationships. Finally, geopolitical changes and regional differences may impact the results, suggesting the need for future studies to consider more detailed temporal and geographic aspects.

Future research could also explore how other macroeconomic variables, such as inflation and interest rates, affect returns in the clean energy market. Regional influence can also be analyzed to capture local and global differences. Another important aspect would be integrating emerging technologies such as AI, blockchain, and IoT to evaluate market performance. Additionally, future research should investigate the temporal evolution of the relationship between GPR, innovation, and returns. Finally, it is essential to study the impact of government policies and examine the differences between short- and long-term effects.

## Supporting information

S1 Appendix Defense industry companies analyzed (source: SIRPI). Note: Companies are ordered by arms revenue in 2022 (million USD).
(DOCX)

S2 Appendix Summary statistics of selected defense stocks (source: Authors’ own compilation). Descriptive statistics are calculated based on input data collected from January 1, 2014, to March 30, 2024, providing an overview of data distribution and characteristics.
(DOCX)

S3 Appendix Plot of daily stock return (source: Authors’ compilation). Note: The horizontal axis covers the period January 1, 2014, to March 29, 2024. The vertical axis represents the daily log return.
(TIFF)

S4 Appendix Wavelet coherence (source: Authors’ compilation). Note: The OX-axis represents time (in years), while the vertical axis represents frequency.
(TIFF)

S5 Appendix Coherence level (source: Authors’ compilation). The figures show the levels of consistency between daily return GPR and MSCI.
(DOCX)

S6 Appendix Nonlinear Granger causality results (Source: Authors’ compilation). Note: The nonlinear Granger causality test results between the daily return, GPR, and MSCI are presented.
(DOCX)

S1 Data (XLSX)
