# Supplementary material for: Impact of geopolitical risks and innovation on global defense stock return
Source: PLoS One. 2025 Feb 21;20(2):e0312155. doi: 10.1371/journal.pone.0312155 (PMC11844836; doi:10.1371/journal.pone.0312155)
Supplement: S1 Appendix — Note: Companies are ordered by arms revenue in 2022 (million USD). (DOCX) [file pone.0312155.s001.docx]

| Rank | Company | Symbol | Country | Arms revenue in 2022 (million USD) |
| --- | --- | --- | --- | --- |
| 1 | Lockheed Martin Co. | LMT | US | 59,390 |
| 2 | Raytheon Technologies Co. BDR | RYTT34 | US | 39,570 |
| 3 | Northrop Grumman Co. | NOC | US | 32,300 |
| 4 | Boeing Co. | BA | US | 29,300 |
| 5 | General Dynamics Co. | GD | US | 28,320 |
| 6 | BAE Systems PLC | BAES | UK | 26,900 |
| 7 | NORINCO International Cooperation Ltd. | 000065 | China | 22,060 |
| 8 | AVIC Aircraft Co. Ltd. | 000768 | China | 20,620 |
| 9 | China Aerospace Times Electronics Co. Ltd. | 600879 | China | 19,560 |
| 10 | CETC Cyberspace Security Technology Co. Ltd. | 002268 | China | 15,080 |
| 11 | L3Harris Technologies Inc. | LHX | US | 12,630 |
| 12 | Leonardo SpA | LDOF | Italy | 12,470 |
| 13 | Airbus Group SE | AIR | France | 12,090 |
| 14 | CSSC Offshore & Marine Engineering Group Ltd. | 600685 | China | 10,440 |
| 15 | Thales Group | TCFP | France | 9,420 |
| 16 | Huntington Ingalls Industries Inc. | HII | US | 8,750 |
| 17 | Leidos Holdings Inc. | LDOS | US | 8,240 |
| 18 | Booz Allen Hamilton Holding | BAH | US | 5,900 |
| 19 | Dassault Aviation SA | AM | France | 5,070 |
| 20 | Elbit Systems Ltd. | ESLT | Israel | 4,960 |
| 21 | Rolls–Royce Holdings PLC | RR | UK | 4,930 |
| 22 | CACI International Inc. | CACI | US | 4,820 |
| 23 | Honeywell International Inc. | HON | US | 4,630 |
| 24 | Rheinmettal AG | RHMG | Germany | 4,550 |
| 25 | General Electric Company | GE | US | 4,410 |
| 26 | KBR Inc. | KBR | US | 4,270 |
| 27 | Safran SA Company | SAF | France | 4,200 |
| 28 | Israel Aerospace Industries Ltd. | ILARSP4=TA | Israel | 4,100 |
| 29 | Science Applications International | SAIC | US | 3,780 |
| 30 | SAAB | SAABBs | Sweden | 3,700 |
| 31 | Babcock International Group PLC | BAB | UK | 3,680 |
| 32 | Hindustan Aeronautics Ltd. | HIAE | India | 3,460 |
| 33 | Rafael Holdings Inc. | RFL | Israel | 3,380 |
| 34 | Mitsubishi Heavy Industries Ltd. | 7011 | Japan | 3,250 |
| 35 | Textron Inc. | TXT | US | 2,910 |
| 36 | Fincantieri SpA | FCT | Italy | 2,820 |
| 37 | CEA Industries Inc. | CEAD | France | 2,790 |
| 38 | Hanwha Aerospace Co. Ltd. | 012450 | South Korea | 2,780 |
| 39 | V2X Inc. | VVX | US | 2,520 |
| 40 | Transdigm Group Inc. | TDG | US | 2,330 |
| 41 | Parker–Hannifin Co. | PH | US | 2,270 |
| 42 | Singapore Tech Engineering Ltd. | STEG | Singapore | 2,180 |
| 43 | Oshkosh Co. | OSK | US | 2,140 |
| 44 | Jacobs Engineering Group Inc. | J | US | 2,090 |
| 45 | Teledyne Technologies Inc. | TDY | US | 2,020 |
| 46 | Aselsan Elektronik Sanayi ve Ticaret AS | ASELS | Türkiye | 2,020 |
| 47 | CNNC International Ltd. | 2302 | China | 1,940 |
| 48 | Thyssenkrupp AG | TKAG | Germany | 1,930 |
| 49 | Bharat Electronics Ltd. | BAJE | India | 1,920 |
| 50 | Serco Group | SRP | UK | 1,850 |
| 51 | Kawasaki Heavy Industries Ltd. | 7012 | Japan | 1,830 |
| 52 | LIG Nex1 Co. Ltd. | 079550 | South Korea | 1,720 |
| 53 | BWX Technologies Inc. | BWXT | US | 1,700 |
| 54 | Hensoldt Ag | HAGG | Germany | 1,660 |
| 55 | Qinetiq Group PLC | QQ | UK | 1,620 |
| 56 | Pan Global Resources Inc. | PGZ | Poland | 1,600 |
| 57 | Korea Aerospace | 047810 | South Korea | 1,550 |
| 58 | Parsons Co. | PSN | US | 1,540 |
| 59 | Eaton Co. PLC | ETN | US | 1,520 |
| 60 | CAE Inc. | CAE | Canada | 1,420 |
| 61 | Curtiss–Wright Co. | CW | US | 1,390 |
| 62 | Moog Inc. | MOGa | US | 1,280 |
| 63 | Fujitsu General Ltd. | 6755 | Japan | 1,270 |
| 64 | Kongsberg Gruppen ASA | KOG | Norway | 1,230 |
| 65 | Amphenol Co. | APH | US | 1,140 |
| 66 | Melrose Industries PLC | MRON | UK | 1,060 |
| 67 | Mazagon Dock Shipbuilders Ltd. | MAZG | India | 1,000 |
| 68 | Austal Ltd. | ASB | Australia | 980 |
| 69 | Mercury Systems Inc. | MRCY | US | 960 |
| 70 | Ball Co. | BALL | US | 930 |
| 71 | Howmet Aerospace Inc. | HWM | US | 920 |
| 72 | TTM Technologies Inc. | TTMI | US | 860 |
| 73 | Heico Co. | HEI | US | 860 |
| 74 | Hyundai–Rotem | 064350 | South Korea | 820 |
| 75 | IHI Co. | 7013 | Japan | 790 |
