# Supplementary material for: Impact of geopolitical risks and innovation on global defense stock return
Source: PLoS One. 2025 Feb 21;20(2):e0312155. doi: 10.1371/journal.pone.0312155 (PMC11844836; doi:10.1371/journal.pone.0312155)
Supplement: S2 Appendix — Descriptive statistics are calculated based on input data collected from January 1, 2014, to March 30, 2024, providing an overview of data distribution and characteristics. (DOCX) [file pone.0312155.s002.docx]

| Company | **Mean** | **Maximum** | **Minimum** | **Std. Dev.** | **Skewness** | **Kurtosis** | **Jarque–Bera** | **Probability** |
| --- | --- | --- | --- | --- | --- | --- | --- | --- |
| LMT | 0.0004 | 0.1019 | -0.1365 | 0.0138 | -0.6008 | 18.669 | 27055.01 | 0.000 |
| RYTT34 | 0.0004 | 0.3494 | -0.2936 | 0.0205 | 1.0782 | 73.2663 | 48678.75 | 0.000 |
| NOC | 0.0005 | 0.1222 | -0.1069 | 0.0150 | -0.0504 | 11.4595 | 7840.297 | 0.000 |
| BA | 0.0001 | 0.2176 | -0.2724 | 0.0245 | -0.5310 | 22.5863 | 42146.50 | 0.000 |
| GD | 0.0004 | 0.0875 | -0.1157 | 0.0137 | -0.3680 | 10.0950 | 5573.613 | 0.000 |
| BAES | 0.0004 | 0.10008 | -0.0870 | 0.0142 | -0.1370 | 8.4046 | 3207.972 | 0.000 |
| 000065 | 0.0001 | 0.1028 | -0.5109 | 0.0318 | -2.2996 | 39.1881 | 14577.09 | 0.000 |
| 000768 | 0.0003 | 0.1122 | -0.1055 | 0.0277 | -0.0889 | 6.3889 | 1261.545 | 0.000 |
| 600879 | -0.0001 | 0.0959 | -0.7989 | 0.0277 | -9.2402 | 65.0544 | 75599.13 | 0.000 |
| 002268 | 0.0001 | 0.0956 | -0.5753 | 0.0328 | -2.0369 | 39.9476 | 15135.64 | 0.000 |
| LHX | 0.0004 | 0.1121 | -0.1543 | 0.0156 | -0.1643 | 14.6922 | 14987.18 | 0.000 |
| LDOF | 0.0005 | 0.1507 | -0.2503 | 0.0231 | -0.7728 | 16.7208 | 20884.26 | 0.000 |
| AIR | 0.0004 | 0.1861 | -0.2507 | 0.0220 | -0.4574 | 19.3936 | 29531.13 | 0.000 |
| 600685 | 0.0001 | 0.0955 | -0.1554 | 0.0300 | 0.0241 | 6.2502 | 1157.479 | 0.000 |
| TCFP | 0.0004 | 0.1285 | -0.1194 | 0.0160 | 0.1628 | 10.4135 | 6032.073 | 0.000 |
| HII | 0.0004 | 0.1080 | -0.1311 | 0.0169 | -0.3939 | 9.6524 | 4915.748 | 0.000 |
| LDOS | 0.0005 | 0.1023 | -0.2035 | 0.0172 | -1.3785 | 21.4815 | 38248.74 | 0.000 |
| BAH | 0.0008 | 0.1273 | -0.2093 | 0.0161 | -1.1974 | 22.1991 | 41006.24 | 0.000 |
| AM | 0.0003 | 0.1152 | -0.1213 | 0.0180 | -0.0532 | 8.2232 | 2989.751 | 0.000 |
| ESLT | 0.0004 | 0.1443 | -0.1116 | 0.0161 | -0.1037 | 9.3648 | 4442.418 | 0.000 |
| RR | -0.0004 | 0.3629 | -1.0897 | 0.0362 | -9.8383 | 32.1829 | 11062.85 | 0.000 |
| CACI | 0.0006 | 0.1401 | -0.1492 | 0.0164 | -0.1575 | 14.7468 | 15126.28 | 0.000 |
| HON | 0.0003 | 0.1403 | -0.1288 | 0.0140 | -0.1989 | 15.6587 | 17570.67 | 0.000 |
| RHMG | 0.0009 | 0.2215 | -0.1254 | 0.0218 | 0.4798 | 12.4880 | 9962.177 | 0.000 |
| GE | 0.0001 | 0.1374 | -0.1644 | 0.0210 | -0.1213 | 10.4013 | 6007.077 | 0.000 |
| KBR | 0.0002 | 0.1682 | -0.2600 | 0.0237 | -1.0960 | 19.7001 | 31076.9 | 0.000 |
| SAF | 0.0005 | 0.1900 | -0.2597 | 0.0204 | -0.6999 | 27.8045 | 67611.98 | 0.000 |
| ILARSP4=TA | 0.0001 | 0.0227 | -0.0289 | 0.0020 | -3.0600 | 68.9056 | 41820.37 | 0.000 |
| SAIC | 0.0005 | 0.1717 | -0.2010 | 0.0192 | -1.3862 | 22.4112 | 42116.91 | 0.000 |
| SAABBs | 0.0006 | 0.1423 | -0.1453 | 0.0191 | -0.0724 | 12.2480 | 9371.068 | 0.000 |
| BAB | -0.0003 | 0.2775 | -0.1790 | 0.0212 | 0.4320 | 20.532 | 33755.16 | 0.000 |
| HIAE | 0.0011 | 0.1593 | -0.1527 | 0.0221 | 0.8241 | 11.1654 | 4455.447 | 0.000 |
| RFL | -0.0006 | 0.2623 | -1.3162 | 0.0540 | -9.1356 | 23.5444 | 33502.56 | 0.000 |
| 7011 | 0.0003 | 2.2660 | -2.2863 | 0.0653 | -0.4414 | 1118.79 | 13608.41 | 0.000 |
| TXT | 0.0003 | 0.1501 | -0.1878 | 0.0205 | -0.4351 | 14.6967 | 15069.84 | 0.000 |
| FCT | 0.0001 | 0.1889 | -0.1706 | 0.0236 | 0.1014 | 11.2707 | 7129.795 | 0.000 |
| CEAD | -0.0024 | 1.1867 | -0.6556 | 0.0785 | 2.0620 | 33.8784 | 10420.57 | 0.000 |
| 012450 | 0.0005 | 0.1862 | -0.2377 | 0.0272 | 0.1827 | 10.3702 | 5965.003 | 0.000 |
| VVX | 0.0003 | 0.3004 | -0.5956 | 0.0309 | -2.7387 | 75.3693 | 53682.77 | 0.000 |
| TDG | 0.0009 | 0.2183 | -0.2481 | 0.0204 | -0.6398 | 27.5405 | 66149.71 | 0.000 |
| PH | 0.0005 | 0.1690 | -0.1825 | 0.0188 | -0.4597 | 15.7386 | 17868.25 | 0.000 |
| STEG | 0.0001 | 0.0840 | -0.1044 | 0.0118 | -0.2660 | 10.7919 | 6681.709 | 0.000 |
| OSK | 0.0003 | 0.1940 | -0.1487 | 0.0212 | 0.0044 | 9.9624 | 5310.097 | 0.000 |
| J | 0.0003 | 0.0978 | -0.1290 | 0.0169 | -0.2620 | 9.3518 | 4449.629 | 0.000 |
| TDY | 0.0005 | 0.1250 | -0.2598 | 0.0168 | -1.5683 | 30.5297 | 84097.89 | 0.000 |
| ASELS | 0.0015 | 0.5885 | -0.4941 | 0.0282 | 1.2150 | 11.1535 | 12819.76 | 0.000 |
| CNNC | 0.0001 | 0.4832 | -0.2759 | 0.0342 | 2.0984 | 35.0860 | 11470.42 | 0.000 |
| TKAG | -0.0004 | 0.2477 | -0.1999 | 0.0281 | -0.0600 | 12.2087 | 9290.811 | 0.000 |
| BAJE | 0.0011 | 0.1645 | -0.1817 | 0.0228 | 0.0772 | 8.9012 | 3817.317 | 0.000 |
| SRP | -0.0002 | 0.1531 | -0.3885 | 0.0223 | -2.3836 | 49.3357 | 23767.59 | 0.000 |
| 7012 | 0.0009 | 2.2671 | -0.1161 | 0.0492 | 37.1050 | 179.363 | 32154.08 | 0.000 |
| 079550 | 0.0003 | 0.2617 | -0.1925 | 0.02727 | 0.52170 | 10.6317 | 5386.879 | 0.000 |
| BWXT | 0.0005 | 0.1369 | -0.2711 | 0.0166 | -1.6005 | 37.5295 | 13172.80 | 0.000 |
| HAGG | 0.0014 | 0.3546 | -0.2047 | 0.0280 | 2.1023 | 36.2974 | 42333.89 | 0.000 |
| QQ | 0.0001 | 0.1059 | -0.1413 | 0.0159 | -0.0608 | 12.9532 | 10853.64 | 0.000 |
| PGZ | 0.0001 | 1.3862 | -0.6931 | 0.0996 | 2.2465 | 52.7224 | 27303.35 | 0.000 |
| 047810 | 0.0002 | 0.1639 | -0.3538 | 0.0247 | -1.0982 | 23.7214 | 47563.10 | 0.000 |
| PSN | 0.0008 | 0.1130 | -0.1599 | 0.0204 | -0.7591 | 12.7671 | 5125.258 | 0.000 |
| ETN | 0.0005 | 0.2091 | -0.1274 | 0.0171 | 0.1787 | 17.5065 | 23065.94 | 0.000 |
| CAE | 0.0002 | 0.1673 | -0.2402 | 0.0205 | -0.9948 | 29.9602 | 80054.41 | 0.000 |
| CW | 0.0005 | 0.1205 | -0.1972 | 0.0177 | -0.5789 | 14.9338 | 15747.52 | 0.000 |
| MOGa | 0.0003 | 0.1655 | -0.2338 | 0.0217 | -0.5876 | 17.8658 | 24359.29 | 0.000 |
| 6755 | 0.0001 | 0.14426 | -0.1360 | 0.0196 | 0.2068 | 9.5303 | 4690.218 | 0.000 |
| KOG | 0.0006 | 0.1209 | -0.2089 | 0.0181 | -0.6630 | 15.6705 | 17778.82 | 0.000 |
| APH | 0.0006 | 0.0963 | -0.1525 | 0.0145 | -0.7266 | 12.6095 | 10346.80 | 0.000 |
| MRON | 0.0006 | 0.3772 | -0.1936 | 0.025 | 1.1826 | 28.1758 | 70042.67 | 0.000 |
| MAZG | 0.0026 | 0.18028 | -0.1223 | 0.0294 | 1.1309 | 8.3112 | 1237.216 | 0.000 |
| ASB | 0.0003 | 0.2281 | -0.2935 | 0.0263 | -0.4059 | 18.5366 | 26514.11 | 0.000 |
| MRCY | 0.0003 | 0.1755 | -0.2067 | 0.0251 | -0.2016 | 13.7882 | 12767.07 | 0.000 |
| BALL | 0.0003 | 0.1257 | -0.2055 | 0.0170 | -0.2895 | 17.5426 | 23203.64 | 0.000 |
| HWM | 0.0008 | 0.233 | -0.2311 | 0.0265 | -0.3891 | 17.4771 | 13469.98 | 0.000 |
| TTMI | 0.0002 | 0.2042 | -0.2050 | 0.0247 | -0.0727 | 15.347 | 16702.08 | 0.000 |
| HEI | 0.0007 | 0.1183 | -0.1788 | 0.0185 | -0.1694 | 11.4177 | 7774.463 | 0.000 |
| 064350 | 0.0001 | 0.2623 | -0.2130 | 0.0291 | 0.8579 | 15.7073 | 18011.03 | 0.000 |
| 7013 | 0.0008 | 2.2883 | -0.2261 | 0.0511 | 33.971 | 52.0351 | 25424.52 | 0.000 |
