# Supplementary figures and images for: Impact of geopolitical risks and innovation on global defense stock return
Source: PLoS One. 2025 Feb 21;20(2):e0312155. doi: 10.1371/journal.pone.0312155 (PMC11844836; doi:10.1371/journal.pone.0312155)

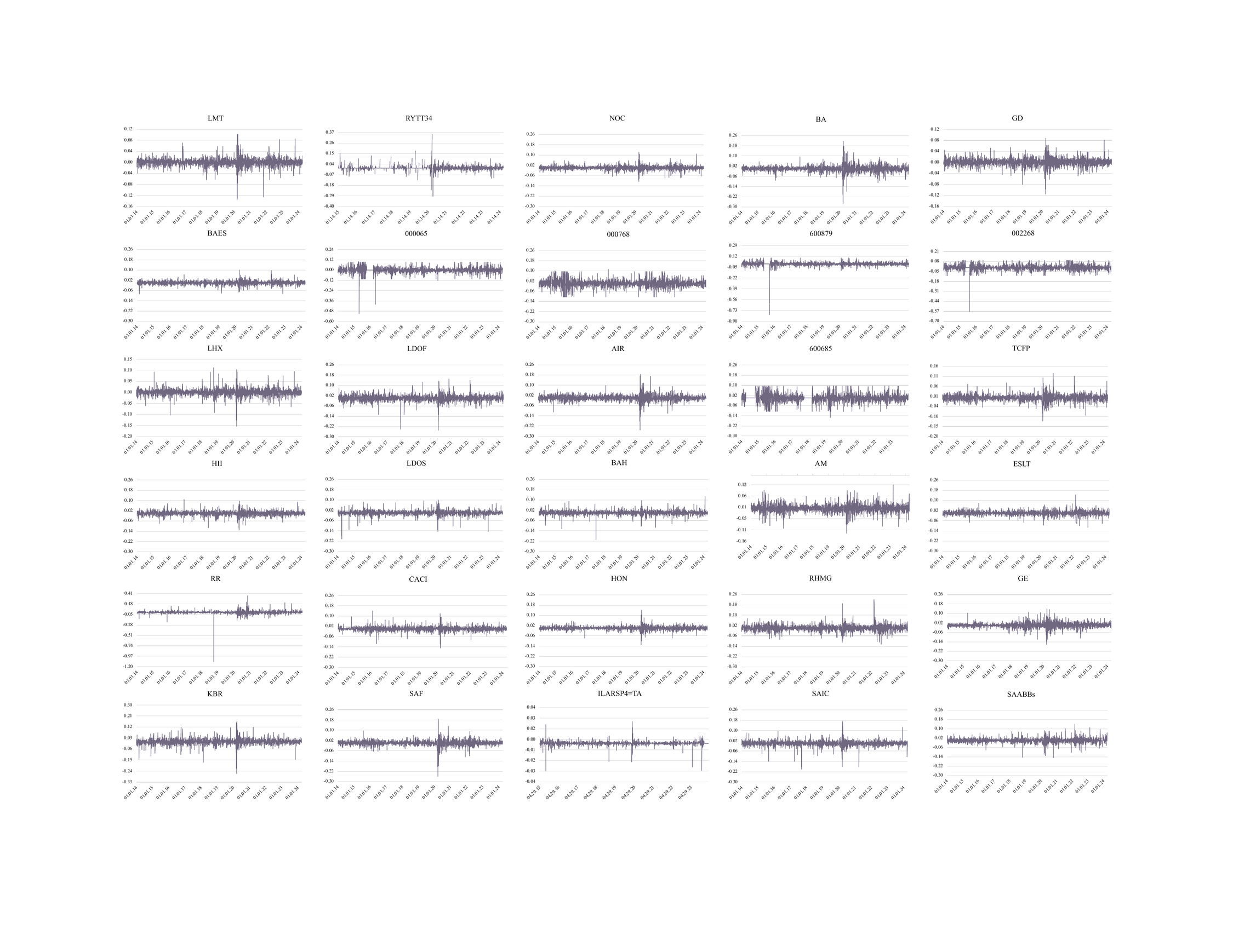

Supplement: S3 Appendix — Note: The horizontal axis covers the period January 1, 2014, to March 29, 2024. The vertical axis represents the daily log return. (TIFF) [file pone.0312155.s003.tiff]

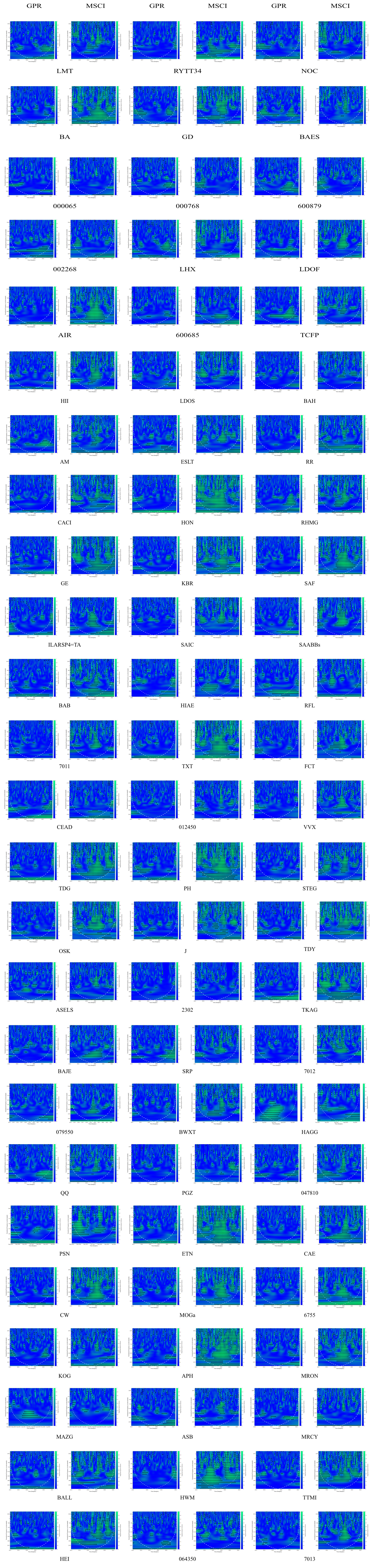

Supplement: S4 Appendix — Note: The OX-axis represents time (in years), while the vertical axis represents frequency. (TIFF) [file pone.0312155.s004.tiff]
