# Supplementary material for: Impact of geopolitical risks and innovation on global defense stock return
Source: PLoS One. 2025 Feb 21;20(2):e0312155. doi: 10.1371/journal.pone.0312155 (PMC11844836; doi:10.1371/journal.pone.0312155)
Supplement: S5 Appendix — The figures show the levels of consistency between daily return GPR and MSCI. (DOCX) [file pone.0312155.s005.docx]

| Rank | Symbol | Country | Coherence level | Stock return and GPR index level coherence | | | Stock return and MSCI index level coherence | | |
| --- | --- | --- | --- | --- | --- | --- | --- | --- | --- |
|  |  |  |  | Small | Medium | High | Small | Medium | High |
| 1 | LMT | US | Medium | √ | √ |  |  | √ | √ |
| 2 | RYTT | US | Low |  |  |  | √ | √ |  |
| 3 | NOC | US | Medium | √ | √ |  |  | √ | √ |
| 4 | BA | US | Medium | √ | √ |  | √ | √ | √ |
| 5 | GD | US | Medium |  | √ |  |  | √ |  |
| 6 | BAES | UK | Strong | √ | √ |  |  | √ |  |
| 7 | 000065 | China | Low |  |  |  |  |  |  |
| 8 | 000768 | China | Medium |  | √ |  |  | √ |  |
| 9 | 600879 | China | Medium |  | √ |  |  | √ |  |
| 10 | 002268 | China | Strong | √ | √ |  | √ | √ |  |
| 11 | LHX | US | Strong | √ | √ |  |  | √ | √ |
| 12 | LDOF | Italy | Medium |  | √ |  |  | √ |  |
| 13 | AIR | France | Medium | √ |  |  | √ | √ | √ |
| 14 | 600685 | China | Low |  |  |  | √ |  |  |
| 15 | TCFP | France | Strong |  | √ |  |  | √ |  |
| 16 | HII | US | Low |  |  |  | √ | √ | √ |
| 17 | LDOS | US | Medium |  | √ |  |  | √ | √ |
| 18 | BAH | US | Low |  |  |  |  |  |  |
| 19 | AM | France | Medium |  | √ |  | √ | √ |  |
| 20 | ESLT | Israel | Medium | √ | √ |  | √ | √ |  |
| 21 | RR | UK | Medium | √ |  |  |  | √ | √ |
| 22 | CACI | US | Low |  |  |  | √ | √ | √ |
| 23 | HON | US | Medium | √ |  |  | √ | √ | √ |
| 24 | RHMG | Germany | Strong | √ | √ |  | √ | √ |  |
| 25 | GE | US | Medium | √ |  |  | √ | √ | √ |
| 26 | KBR | US | Medium | √ |  |  | √ | √ | √ |
| 27 | SAF | France | Low |  |  |  | √ | √ | √ |
| 28 | ILARSP4=TA | Israel | Medium |  | √ |  | √ | √ |  |
| 29 | SAIC | US | Medium |  | √ |  |  | √ | √ |
| 30 | SAABBs | Sweden | Medium | √ | √ |  |  | √ |  |
| 31 | BAB | UK | Medium | √ |  |  | √ | √ | √ |
| 32 | HIAE | India | Medium |  | √ |  |  | √ |  |
| 33 | RFL | Israel | Strong | √ |  |  | √ | √ |  |
| 34 | 7011 | Japan | Low |  |  |  |  | √ |  |
| 35 | TXT | US | Medium | √ |  |  | √ | √ | √ |
| 36 | FCT | Italy | Low |  |  |  |  | √ |  |
| 37 | CEAD | France | Strong | √ | √ |  | √ | √ |  |
| 38 | 012450 | South Korea | Low |  |  |  |  | √ |  |
| 39 | VVX | US | Low |  |  |  |  | √ |  |
| 40 | TDG | US | Strong | √ | √ |  | √ | √ | √ |
| 41 | PH | US | Medium | √ | √ |  | √ | √ | √ |
| 42 | STEG | Singapore | Low |  |  |  |  | √ |  |
| 43 | OSK | US | Low |  |  |  |  | √ | √ |
| 44 | J | US | Medium | √ | √ |  | √ | √ | √ |
| 45 | TDY | US | Medium | √ | √ |  | √ | √ | √ |
| 46 | ASELS | Türkiye | Medium |  | √ |  |  | √ |  |
| 47 | 2302 | China | Low |  |  |  |  |  |  |
| 48 | TKAG | Germany | Strong | √ |  |  | √ | √ | √ |
| 49 | BAJE | India | Low |  |  |  |  | √ |  |
| 50 | SRP | UK | Strong | √ | √ |  |  | √ |  |
| 51 | 7012 | Japan | Low |  |  |  |  | √ |  |
| 52 | 079550 | South Korea | Medium |  | √ |  | √ | √ |  |
| 53 | BWXT | US | Low |  |  |  |  | √ | √ |
| 54 | HAGG | Germany | Strong | √ | √ |  | √ | √ |  |
| 55 | QQ | UK | Strong |  | √ |  |  | √ |  |
| 56 | PGZ | Poland | Low |  |  |  |  | √ |  |
| 57 | 047810 | South Korea | Medium |  | √ |  |  | √ |  |
| 58 | PSN | US | Strong | √ |  |  |  | √ | √ |
| 59 | ETN | US | Medium | √ | √ |  | √ | √ | √ |
| 60 | CAE | Canada | Medium | √ |  |  | √ | √ |  |
| 61 | CW | US | Medium | √ |  |  | √ | √ | √ |
| 62 | MOGa | US | Medium | √ | √ |  |  | √ | √ |
| 63 | 6755 | Japan | Low |  |  |  | √ | √ | √ |
| 64 | KOG | Norway | Medium |  | √ |  | √ | √ |  |
| 65 | APH | US | Medium |  | √ |  | √ | √ | √ |
| 66 | MRON | UK | Medium | √ | √ |  | √ | √ | √ |
| 67 | MAZG | India | Medium |  | √ |  |  |  |  |
| 68 | ASB | Australia | Medium | √ |  |  |  | √ |  |
| 69 | MRCY | US | Medium |  | √ |  |  | √ |  |
| 70 | BALL | US | Medium | √ | √ |  | √ | √ | √ |
| 71 | HWM | US | Medium |  | √ |  | √ | √ | √ |
| 72 | TTMI | US | Low |  |  |  | √ | √ | √ |
| 73 | HEI | US | Medium | √ |  |  | √ | √ | √ |
| 74 | 064350 | South Korea | Medium | √ |  |  |  | √ |  |
| 75 | 7013 | Japan | Low |  |  |  |  | √ |  |
