# Supplementary material for: Impact of geopolitical risks and innovation on global defense stock return
Source: PLoS One. 2025 Feb 21;20(2):e0312155. doi: 10.1371/journal.pone.0312155 (PMC11844836; doi:10.1371/journal.pone.0312155)
Supplement: S6 Appendix — Note: The nonlinear Granger causality test results between the daily return, GPR, and MSCI are presented. (DOCX) [file pone.0312155.s006.docx]

| Index | LMT | GPR | MSCI | Index | RYTT34 | GPR | MSCI | Index | NOC | GPR | MSCI |
| --- | --- | --- | --- | --- | --- | --- | --- | --- | --- | --- | --- |
| LMT | - | 4.0233 | 7.9037 | RYTT34 | - | 2.2150 | 21.248 | NOC | - | 4.8614 | 8.3647 |
| GPR | 1.9718 | - | 3.4609 | GPR | 1.8363 | - | 3.5449 | GPR | 0.2750 | - | 3.4629 |
| MSCI | 27.067 | 7.1192 | - | MSCI | 40.410 | 6.1164 | - | MSCI | 20.242 | 8.0937 | - |
| Index | BA | GPR | MSCI | Index | GD | GPR | MSCI | Index | BAES | GPR | MSCI |
| BA | - | 0.4611 | 18.430 | GD | - | 3.9295 | 2.8874 | BAES | - | 3.1396 | 2.9769 |
| GPR | 0.0665 | - | 3.5280 | GPR | 1.9642 | - | 3.2631 | GPR | 3.7510 | - | 3.1780 |
| MSCI | 13.705 | 5.6042 | - | MSCI | 31.354 | 3.2162 | - | MSCI | 14.561 | 7.0749 | - |
| Index | 000065 | GPR | MSCI | Index | 000768 | GPR | MSCI | Index | 600879 | GPR | MSCI |
| 000065 | - | 9.8299 | 2.7592 | 768 | - | 2.9347 | 1.6695 | 600879 | - | 1.0368 | 2.1041 |
| GPR | 5.8216 | - | 3.5842 | GPR | 2.0141 | - | 3.5896 | GPR | 3.3655 | - | 3.2985 |
| MSCI | 8.2318 | 6.2429 | - | MSCI | 10.618 | 7.0127 | - | MSCI | 4.0422 | 6.2875 | - |
| Index | 002268 | GPR | MSCI | Index | LHX | GPR | MSCI | Index | LDOF | GPR | MSCI |
| 002268 | - | 1.1237 | 2.6422 | LHX | - | 7.9627 | 4.2941 | LDOF | - | 3.7172 | 12.006 |
| GPR | 1.5162 | - | 3.3905 | GPR | 0.9886 | - | 3.2362 | GPR | 6.0228 | - | 3.1504 |
| MSCI | 18.663 | 6.8928 | - | MSCI | 16.383 | 8.1089 | - | MSCI | 12.762 | 6.2821 | - |
| Index | AIR | GPR | MSCI | Index | 600685 | GPR | MSCI | Index | TCFP | GPR | MSCI |
| AIR | - | 7.2108 | 11.449 | 600685 | - | 2.3985 | 0.9905 | TCFP | - | 6.9276 | 13.217 |
| GPR | 3.1294 | - | 3.2034 | GPR | 2.7858 | - | 3.3939 | GPR | 8.6471 | - | 3.2578 |
| MSCI | 19.264 | 2.2422 | - | MSCI | 21.063 | 6.6431 | - | MSCI | 12.619 | 7.2662 | - |
| Index | HII | GPR | MSCI | Index | LDOS | GPR | MSCI | Index | BAH | GPR | MSCI |
| HII | - | 4.0931 | 0.1953 | LDOS | - | 0.1235 | 2.3833 | BAH | - | 2.0894 | 4.3163 |
| GPR | 3.4905 | - | 3.3722 | GPR | 3.5932 | - | 3.4676 | GPR | 6.2617 | - | 3.5439 |
| MSCI | 8.2182 | 5.5440 | - | MSCI | 8.1391 | 5.3133 | - | MSCI | 8.5579 | 8.2603 | - |
| Index | AM | GPR | MSCI | Index | ESLT | GPR | MSCI | Index | RR | GPR | MSCI |
| AM | - | 0.9079 | 10.471 | ESLT | - | 10.780 | 7.281 | RR | - | 7.4625 | 6.3406 |
| GPR | 4.4579 | - | 3.4317 | GPR | 2.3359 | - | 3.4368 | GPR | 2.3666 | - | 3.2695 |
| MSCI | 21.058 | 6.9434 | - | MSCI | 11.606 | 11.858 | - | MSCI | 14.794 | 4.4912 | - |
| Index | CACI | GPR | MSCI | Index | HON | GPR | MSCI | Index | RHMG | GPR | MSCI |
| CACI | - | 3.0746 | 3.3613 | HON | - | 4.4885 | 2.2088 | RHMG | - | 15.889 | 13.901 |
| GPR | 7.0753 | - | 3.5687 | GPR | 2.6121 | - | 3.3264 | GPR | 14.637 | - | 3.4233 |
| MSCI | 8.1095 | 7.6513 | - | MSCI | 6.0362 | 1.2372 | - | MSCI | 32.144 | 12.238 | - |
| Index | GE | GPR | MSCI | Index | KBR | GPR | MSCI | Index | SAF | GPR | MSCI |
| GE | - | 0.1530 | 1.7683 | KBR | - | 1.3460 | 9.1993 | SAF | - | 9.1558 | 20.844 |
| GPR | 0.6882 | - | 3.2994 | GPR | 2.1887 | - | 3.3406 | GPR | 2.7870 | - | 2.6859 |
| MSCI | 1.1173 | 4.3446 | - | MSCI | 12.213 | 5.9249 | - | MSCI | 19.309 | 2.0277 | - |
| Index | ILARSP4=TA | GPR | MSCI | Index | SAIC | GPR | MSCI | Index | SAABBs | GPR | MSCI |
| ILARSP4=TA | - | 10.285 | 10.943 | SAIC | - | 6.0244 | 2.0876 | SAABBs | - | 2.5757 | 10.705 |
| GPR | 1.4862 | - | 3.7455 | GPR | 3.5906 | - | 3.3984 | GPR | 8.3691 | - | 3.3161 |
| MSCI | 15.728 | 6.1102 | - | MSCI | 29.678 | 11.579 | - | MSCI | 39.985 | 6.7852 | - |
| Index | BAB | GPR | MSCI | Index | HIAE | GPR | MSCI | Index | RFL | GPR | MSCI |
| BAB | - | 1.4045 | 2.1418 | HIAE | - | 0.6472 | 8.8173 | RFL | - | 1.2733 | 2.8649 |
| GPR | 3.8803 | - | 3.1926 | GPR | 2.1409 | - | 2.5596 | GPR | 3.2201 | - | 2.3852 |
| MSCI | 9.0484 | 5.2690 | - | MSCI | 31.164 | 4.9988 | - | MSCI | 16.274 | 4.3249 | - |
| Index | 7011 | GPR | MSCI | Index | TXT | GPR | MSCI | Index | FCT | GPR | MSCI |
| 7011 | - | 1.7385 | 1.6915 | TXT | - | 0.9129 | 5.2971 | FCT | - | 1.3240 | 24.747 |
| GPR | 1.2240 | - | 3.4722 | GPR | 3.2991 | - | 3.4255 | GPR | 2.8939 | - | 3.9326 |
| MSCI | 8.3356 | 6.4914 | - | MSCI | 10.615 | 3.0264 | - | MSCI | 3.8560 | 5.6036 | - |
| Index | CEAD | GPR | MSCI | Index | 012450 | GPR | MSCI | Index | VVX | GPR | MSCI |
| CEAD | - | 1.1694 | 7.1734 | 012450 | - | 3.2127 | 1.4538 | VVX | - | 3.5356 | 0.9283 |
| GPR | 3.3300 | - | 3.4845 | GPR | 0.9274 | - | 3.4840 | GPR | 4.6377 | - | 3.5783 |
| MSCI | 8.8484 | 6.7385 | - | MSCI | 63.965 | 7.0882 | - | MSCI | 7.3233 | 7.9030 | - |
| Index | TDG | GPR | MSCI | Index | PH | GPR | MSCI | Index | STEG | GPR | MSCI |
| TDG | - | 6.0451 | 18.363 | PH | - | 3.8468 | 10.110 | STEG | - | 1.7117 | 10.376 |
| GPR | 6.3634 | - | 3.1770 | GPR | 0.6166 | - | 3.7675 | GPR | 3.3797 | - | 3.6314 |
| MSCI | 18.166 | 6.7934 | - | MSCI | 5.1158 | 4.6012 | - | MSCI | 161.03 | 6.3610 | - |
| Index | OSK | GPR | MSCI | Index | J | GPR | MSCI | Index | TDY | GPR | MSCI |
| OSK | - | 1.9221 | 2.7830 | J | - | 2.3742 | 4.6934 | TDY | - | 0.1342 | 4.4228 |
| GPR | 1.1016 | - | 3.4065 | GPR | 4.0468 | - | 3.3104 | GPR | 4.1829 | - | 3.5881 |
| MSCI | 11.345 | 4.9318 | - | MSCI | 5.7146 | 5.4109 | - | MSCI | 17.063 | 3.3993 | - |
| Index | ASELS | GPR | MSCI | Index | 2302 | GPR | MSCI | Index | TKAG | GPR | MSCI |
| ASELS | - | 5.4128 | 5.7999 | 2302 | - | 1.5253 | 2.8171 | TKAG | - | 0.2952 | 12.648 |
| GPR | 3.7349 | - | 3.3733 | GPR | 1.1307 | - | 3.3979 | GPR | 2.4020 | - | 3.0518 |
| MSCI | 6.9272 | 6.6608 | - | MSCI | 6.7129 | 6.5056 | - | MSCI | 13.162 | 4.4358 | - |
| Index | BAJE | GPR | MSCI | Index | SRP | GPR | MSCI | Index | 7012 | GPR | MSCI |
| BAJE | - | 1.9726 | 17.302 | SRP | - | 3.3655 | 1.2402 | 7012 | - | 1.8855 | 2.5466 |
| GPR | 0.6775 | - | 3.2821 | GPR | 3.7680 | - | 3.3767 | GPR | 0.8504 | - | 3.4026 |
| MSCI | 26.400 | 6.3309 | - | MSCI | 14.161 | 5.4665 | - | MSCI | 30.901 | 6.3695 | - |
| Index | 079550 | GPR | MSCI | Index | BWXT | GPR | MSCI | Index | HAGG | GPR | MSCI |
| 079550 | - | 1.3128 | 2.9326 | BWXT | - | 1.3223 | 1.4916 | HAGG | - | 21.028 | 7.6190 |
| GPR | 2.0227 | - | 3.4606 | GPR | 2.2411 | - | 3.5662 | GPR | 6.9639 | - | 3.5840 |
| MSCI | 34.102 | 6.2748 | - | MSCI | 9.7176 | 5.1637 | - | MSCI | 5.8317 | 3.3593 | - |
| Index | QQ | GPR | MSCI | Index | PGZ | GPR | MSCI | Index | 047810 | GPR | MSCI |
| QQ | - | 6.8935 | 7.3801 | PGZ | - | 0.5261 | 1.2338 | 047810 | - | 0.8397 | 2.2322 |
| GPR | 2.7717 | - | 3.2236 | GPR | 0.9621 | - | 3.4199 | GPR | 2.3585 | - | 3.4219 |
| MSCI | 19.305 | 6.9830 | - | MSCI | 7.9260 | 6.4181 | - | MSCI | 79.189 | 6.2686 | - |
| Index | PSN | GPR | MSCI | Index | ETN | GPR | MSCI | Index | CAE | GPR | MSCI |
| PSN | - | 7.6594 | 7.9117 | ETN | - | 9.5438 | 2.8332 | CAE | - | 4.6084 | 7.8031 |
| GPR | 4.4075 | - | 3.1066 | GPR | 0.7632 | - | 3.1051 | GPR | 2.9693 | - | 3.1525 |
| MSCI | 6.1365 | 7.2843 | - | MSCI | 14.646 | 3.1974 | - | MSCI | 11.887 | 4.1116 | - |
| Index | CW | GPR | MSCI | Index | MOGa | GPR | MSCI | Index | 6755 | GPR | MSCI |
| CW | - | 0.8239 | 10.821 | MOGa | - | 2.1241 | 10.990 | 6755 | - | 4.4919 | 1.3747 |
| GPR | 1.4177 | - | 2.9328 | GPR | 6.2919 | - | 3.3089 | GPR | 0.4583 | - | 3.5318 |
| MSCI | 9.9055 | 5.3580 | - | MSCI | 14.326 | 6.2077 | - | MSCI | 77.012 | 6.6244 | - |
| Index | KOG | GPR | MSCI | Index | APH | GPR | MSCI | Index | MRON | GPR | MSCI |
| KOG | - | 4.1956 | 3.9483 | APH | - | 2.8311 | 2.0011 | MRON | - | 3.8831 | 4.2834 |
| GPR | 9.4362 | - | 3.2776 | GPR | 3.5873 | - | 3.4211 | GPR | 0.8181 | - | 3.4074 |
| MSCI | 40.527 | 5.1418 | - | MSCI | 11.116 | 2.5915 | - | MSCI | 13.185 | 7.3584 | - |
| Index | MAZG | GPR | MSCI | Index | ASB | GPR | MSCI | Index | MRCY | GPR | MSCI |
| MAZG | - | 1.4774 | 0.8662 | ASB | - | 6.6685 | 6.1108 | MRCY | - | 6.1510 | 11.842 |
| GPR | 1.9245 | - | 3.4125 | GPR | 1.3519 | - | 2.9924 | GPR | 0.7243 | - | 3.5158 |
| MSCI | 12.029 | 2.3828 | - | MSCI | 51.160 | 5.4048 | - | MSCI | 13.799 | 6.5014 | - |
| Index | BALL | GPR | MSCI | Index | HWM | GPR | MSCI | Index | TTMI | GPR | MSCI |
| BALL | - | 1.3575 | 3.6189 | HWM | - | 2.2435 | 6.7618 | TTMI | - | 0.5245 | 2.3553 |
| GPR | 3.1386 | - | 3.2102 | GPR | 0.7811 | - | 2.1907 | GPR | 3.9484 | - | 3.4976 |
| MSCI | 4.9862 | 3.6342 | - | MSCI | 16.856 | 2.2636 | - | MSCI | 16.316 | 5.5024 | - |
| Index | HEI | GPR | MSCI | Index | 064350 | GPR | MSCI | Index | 7013 | GPR | MSCI |
| HEI | - | 5.2202 | 10.605 | 064350 | - | 1.9933 | 1.8093 | 7013 | - | 0.4999 | 2.0043 |
| GPR | 3.4614 | - | 3.5601 | GPR | 0.5964 | - | 3.3349 | GPR | 0.6930 | - | 3.5373 |
| MSCI | 23.900 | 5.5054 | - | MSCI | 54.088 | 5.9277 | - | MSCI | 51.737 | 6.4454 | - |
